# An ESCRT module is required for neuron pruning

**DOI:** 10.1038/srep08461

**Published:** 2015-02-13

**Authors:** Nicolas Loncle, Monica Agromayor, Juan Martin-Serrano, Darren W. Williams

**Affiliations:** 1 MRC Centre for Developmental Neurobiology, King's College London, London, SE1 1UL; 2 Department of Infectious Diseases, Second Floor Borough Wing, Guy's Hospital, London, SE1 9RT

## Abstract

Neural circuits are refined by both functional and structural changes. Structural remodeling by large-scale pruning occurs where relatively long neuronal branches are cut away from their parent neuron and removed by local degeneration. Until now, the molecular mechanisms executing such branch severing events have remained poorly understood. Here, we reveal a role for the Endosomal Sorting Complex Required for Transport (ESCRT) machinery during neuronal remodeling. Our data show that a specific ESCRT pruning module, including members of the ESCRT-I and ESCRT-III complexes, but not ESCRT-0 or ESCRT-II, are required for the neurite scission event during pruning. Furthermore we show that this ESCRT module requires a direct, *in vivo*, interaction between Shrub/CHMP4B and the accessory protein Myopic/HD-PTP.

Neuronal remodeling is a fundamental mechanism that is essential for building a functional nervous system^1^. During development individual neurons grow exuberantly, often generating many more branches than required. A significant number of aberrant or redundant branches are pruned back by branch-specific local degeneration events during a period of circuit maturation^2^,^3^.

In recent years, studies in *Drosophila* have contributed greatly to our understanding of the cellular and molecular mechanisms of large-scale pruning. These studies have revealed that *TGFβ* and Ecdysone signaling pathways initiate pruning and that the Ubiquitin-Proteasome System, caspases, *hdc* and calcium signaling events are required, along with *Kat60L*, *IKK* and *Mical* for pruning to proceed to completion^4^. All of these activities lead up to the point at which a branch is cut away from the parent neuron. How this final scission event is controlled and what cuts the nerve cell membrane is currently unknown.

To address this question we looked at how similar membrane bending and scission events occur within cells. The Endosomal Sorting Complex Required for Transport (ESCRT) machinery has been shown to execute membrane-cutting events during intraluminal vesicular formation, viral budding, membrane repair and cytokinetic abscission^5^,^6^,^7^,^8^,^9^. The ESCRT proteins were first identified in yeast as class E vacuolar sorting (Vps) proteins and they are functionally conserved from yeast to human^10^. The ESCRTs form four heteromeric protein complexes named ESCRT-0, -I, -II, and -III involved in cargo recognition and sorting as well as membrane bending and cutting. Each of these processes requires the sequential assembly of a specific ESCRT module, composed of proteins from different ESCRT complexes, as well as associated adaptor proteins^11^. For example, during final stages of cytokinesis, the ESCRT-I component TSG101 and the Bro-domain adaptor protein Alix, are recruited to the midbody, where they, in turn, recruit the ESCRT-III CHMP4B, a key player in the scission machinery^12^.

Here we explore and identify a requirement for the ESCRTs during the developmental pruning of axons and dendrites in *Drosophila*.

## Results

### A subset of ESCRT proteins are required for pruning

**Figure 1:**
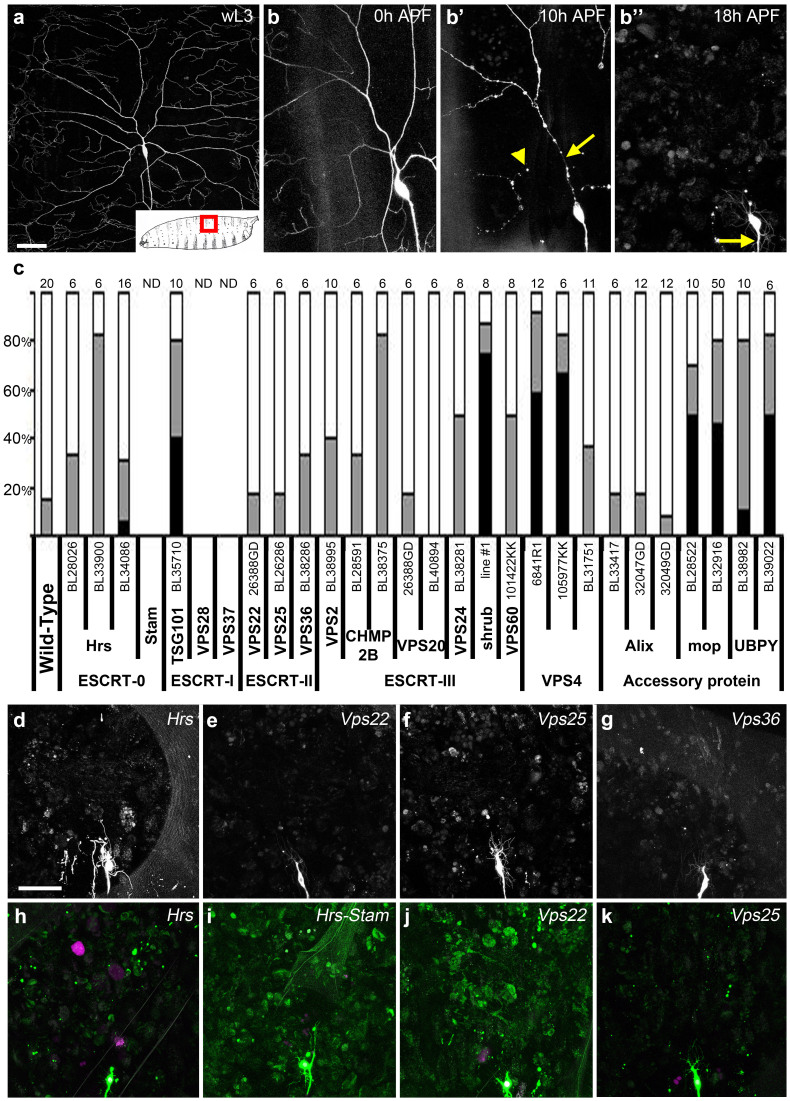
ESCRT-0 and -II are not required for dendrite pruning in the PNS. (a) The class IV dendritic arborizing neuron ddaC is located on the dorsal body wall in each larval segment (box, inset) and elaborates extensive peripheral neurites on epidermal cells. (b) At 0 h after puparium formation (APF) the proximal branches are intact. (b′) At 10 h APF proximal branches are remodeling, some have undergone severing (arrowhead) whilst others have started to thin (arrow). (b″) By 18 h APF most detached dendritic branches have been cleared, the cell body and axon (arrow) remain intact. (c) Results from the screen with the percentage of neurons with; no phenotype (white), clearance defects (gray) and severing defects (black) for each RNAi line tested, with the number of neurons imaged above each column. ND = not determined. (d–g) Results from the RNAi screen showing representative images of a ddaC neuron at 18 h APF expressing RNAi against ESCRT-0 (*Hrs*), and ESCRT-II (*Vps22*, *Vps25*, *Vps36*). For all RNAi tested n = 6 or >6. (h–k) Single-cell ddaC modified MARCM clones at 18 h APF of *Hrs^D28^* (n = 9), *Hrs^D28^-Stam*^2L-2896^ (n = 5), *Vps22^SS6^* (n = 10), and *Vps25^A3^* (n = 10) removed their dendrites as in wild-type. Nucleus reporter red stinger in magenta. Scale bar = 50 μm.

We performed a candidate RNAi screen to knock down multiple representatives from each of the four ESCRT complexes (-0, -I, -II and -III) and ESCRT related proteins in the class IV dendritic arborizing (da) sensory neuron, ddaC (Fig. 1a and b)^13^. These neurons were specifically imaged with the pickpocket-GAL4 driver (ppk1.9-GAL4) at 18 h After Puparium Formation (APF) to assess pruning (Fig. 1b″). We tested twenty-five RNAi lines targeting fifteen of the 18 genes forming the ESCRT machinery in *Drosophila* (Fig. 1c).

**Figure 2:**
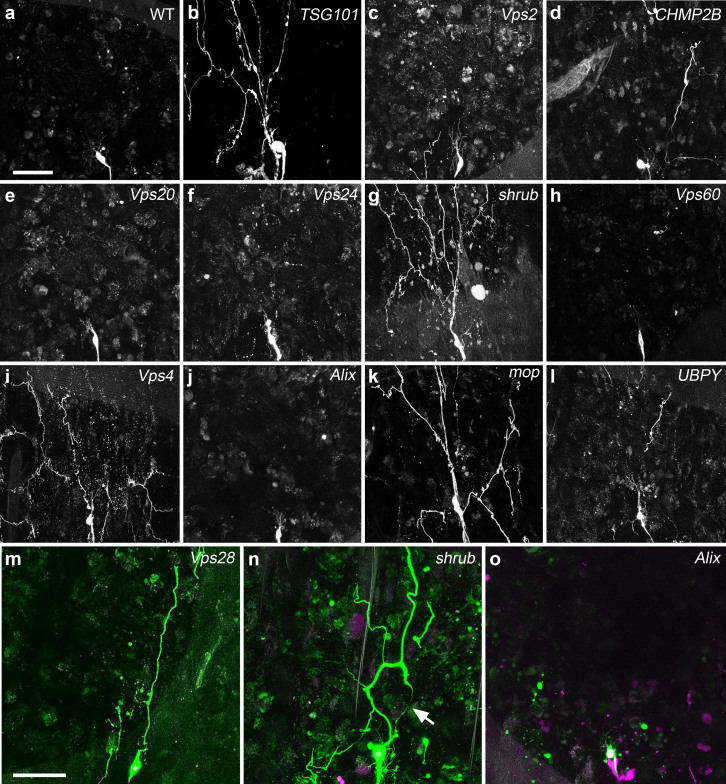
ESCRT-I,-III and accessory proteins are required for dendrite pruning in the PNS. (a–l) Results from the RNAi screen showing representative images of a ddaC neuron at 18 h APF expressing RNAi against ESCRT-I (*TSG101*), ESCRT-III (*Vps2*, *CHMP2B*, *Vps20*, *Vps24*, *shrub* and *Vps60*), *Vps4* and ESCRT-accessory proteins (*Alix*, *mop*, and *Ubpy*). For all RNAi tested n = 6 or >6. (m–o) Single-cell MARCM clones at 18 h APF of *Vps28^B9^* (n = 10) with dendritic debris remaining in field, *shrub^4^* (n = 18) retain dendrites with thin tether (arrowhead) whereas *Alix^LL05494^* clones prune like wild-type (n = 8). Scale bar = 50 μm.

The downregulation of five ESCRT related genes, TSG101, Shrub, Vps4, mop and UBPY, by expressing 9 different RNAi lines, lead to severe disruptions in pruning with dendrites still being attached to the cell body by 18 h APF, resulting from a lack of branch severing (Fig. 1c and 2b, g, i, k and l). These experiments demonstrate the involvement of the ESCRT machinery during pruning in a cell autonomous manner.

The loss of ESCRT-0 subunit *Hrs* led to minor delays in pruning (Fig. 1c) and none of the three subunits of ESCRT-II (*Vps22*, *25* and *36*) displayed a pruning defect (Fig. 1e–g). These data suggested the interesting possibility that only ESCRT proteins belonging to ESCRT-I and -III are required for pruning.

As these experiments were obtained using RNAi based tools, we attempted to confirm these results using a modified MARCM clonal analysis^14^ that allows us to visualize two control neurons alongside the homozygous mutant neurons in the same animal. This MARCM clone analysis allows the cell autonomous requirement of the ESCRT genes to be unequivocally tested. We generated MARCM clones mutant for *Hrs*, *Vps22*, *Vps25* as well as double mutant for both *Hrs* and *Stam* (removing both ESCRT-0 subunits) (Fig. 1h–k). All the homozygous mutant neurons remodeled like wild type neurons, revealing that these ESCRT-0 and ESCRT-II subunits are not required for pruning. These homozygous mutant clones show normal regrowth following pruning, generating numerous filopodia on their emerging growth cones.

Alongside this, MARCM clones against Vps28 (Fig. 2m) confirmed that ESCRT-I is necessary for pruning, as the strong severing defect observed with TSG101 RNAi had suggested (Fig. 1c and 2b). For the ESCRT-III, we focused on Shrub the CHMP4B homolog in flies^15^. Shrub is a key component of the scission machinery and forms the oligomeric helical filament that bends membranes together to allow cutting to take place^16^. MARCM analysis of neurons homozygous mutant for Shrub reveal strong defects in severing, with dendrites still attached to the soma by 18 h APF (Fig. 2n).

Together multiple RNAi knockdown and MARCM clone data with null and strong alleles show that ESCRTs are required, in a cell autonomous manner, for pruning. The requirement of a specific subset of ESCRT including components of ESCRT-I, -III and Vps4 suggest the existence of an ESCRT pruning module.

### Myopic accessory protein is required for pruning

The ESCRT genes are expressed ubiquitously and the activity of their proteins needs to be tightly regulated to allow them to undertake different roles within the cell. One aspect of this regulation is their modular assembly, i.e. the context-specific generation of discrete combinations of ESCRT proteins and their accessory proteins^7^,^12^. The ESCRT-accessory proteins play a key role in recruiting and stabilizing ESCRT subunits. During cytokinesis the ESCRT-III complex is recruited to the midbody by the Bro-domain ESCRT-accessory protein Alix^17^,^18^,^19^. In our screen none of the RNAi reagents targeting Alix lead to defects in pruning (Fig. 2j). To explore this further we generated single cell mutant MARCM clones homozygous for *Alix^LL05494^* and also found no disruption in pruning (n = 8) (Fig. 2o). These data suggest that another accessory protein is required for ESCRT-III mediated severing in pruning neurons. *Myopic* (*mop*), the homolog of the human gene *HD-PTP* is another Bro-domain protein found in the fly genome. Mop is known to be involved in ESCRT-mediated receptor sorting^20^,^21^. When we knocked down the expression of *mop* with two independent RNAi lines, we observed a robust block of dendrite severing in ddaC (Fig. 1c and 2k) revealing it to be a key ESCRT-accessory protein required for pruning.

### Shrub function during pruning requires a direct interaction with the accessory protein Mop

**Figure 3:**
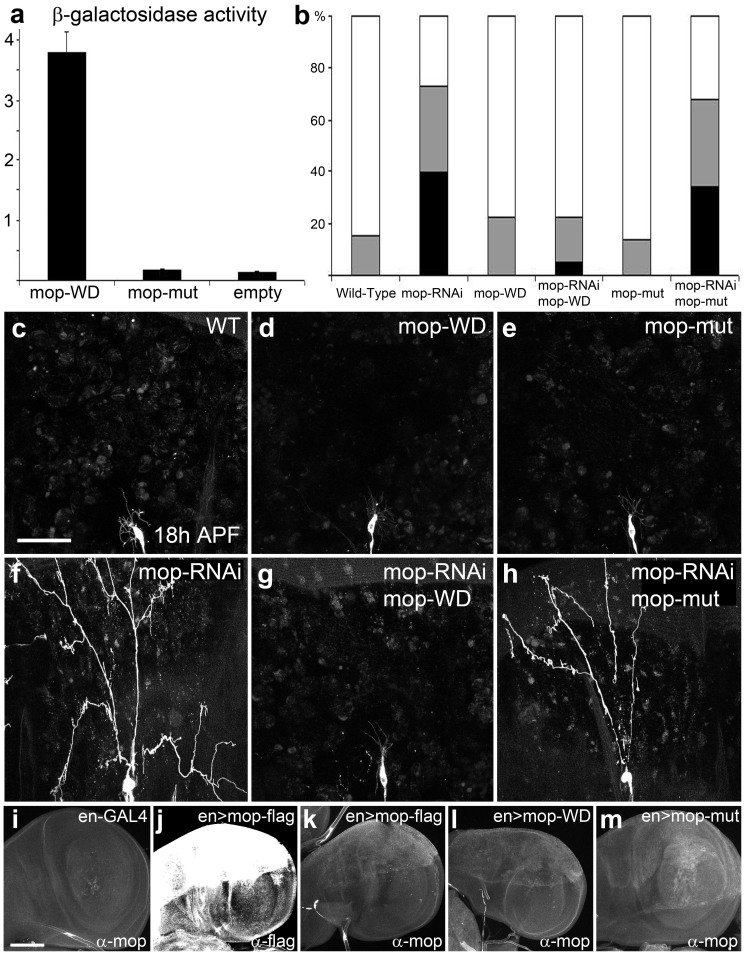
An interaction between Shrub and Mop is required for pruning. (a) A Yeast-two-hybrid assay detects an interaction between Shrub (bait) and Mop Bro-domain. Wild-type Mop Bro-domain (mop-WD) binds to Shrub whereas the mutant Bro-domain (mutations in I201D and V205D) (mop-mut) does not bind. These tests were repeated four times, error bars indicate the standard deviations from the mean of triplicate measurements. (b) Quantification of pruning defect for the *in vivo* *mop* rescue experiments, Black = severing defect, Gray = clearance defect. White = wild-type pruning. N = 48, 40, 40, 37 and 44, respectively. ppk-GAL4 > CD8::GFP shows wild-type pruning at 18 h APF (c) as do ddaC neurons expressing UAS-mop-WD (wild type Bro-domain), n = 40 (d) or UAS-mop-mut (point mutation I201D and V205D in the Bro-domain), n = 37 (e). ddaC neurons expressing mop-RNAi show a robust disruption in severing at 18 h APF, n = 48 (f). mop-RNAi combined with UAS-mop-WD completely rescues the RNAi severing defect, n = 40 (g). mop-RNAi along with UAS-mop-mut results in a robust severing defect, n = 44 (h). Imaginal wing disc expressing of UAS-mop constructs under the control of en-GAL4 (i–m). Comparison between Flag and Mop antibody (j–k) Detection by Mop antibody shows similar levels of expression of UAS-mop-WD and UAS-mop-mut (l–m). Scale bar = 50 μm.

Since *shrub* and *mop* are required for pruning, we next investigated how these two proteins interact with each other and whether a direct interaction between them is necessary for their function. Previous work identified that HD-PTP (the human homologue of *mop*) physically interacts with CHMP4B (the homologue of *shrub*) through the Bro1 domain of HD-PTP and that this interaction can be disrupted by introducing the point mutations L202D and I206D^20^. We confirmed that this interaction is conserved between the *Drosophila* Shrub and Mop using a yeast-two-hybrid assay (Fig. 3a) and that the disruption of the conserved amino acid I201D and V205D within the Bro1 domain of Mop (equivalent to the human L202 and I206) is sufficient to inhibit this interaction. To test if this same interaction is relevant *in vivo* and required for branch severing, we generated transgenic flies expressing wild-type or mutant forms of mop (I201D and V205D) at similar levels (Fig. 3i–m). Each was used in turn to test whether they could rescue pruning in a mop-RNAi background (Fig. 3f). We found wild-type Mop was able to fully rescue the RNAi severing phenotype in ddaC (Fig. 3g) but the Bro-domain mutant Mop could not (Fig. 3h). Together, these results show that branch severing requires a physical interaction between Mop and Shrub, which is dependent on the Bro domain of Mop.

### ESCRT function is required for pruning of other classes of neurons

**Figure 4:**
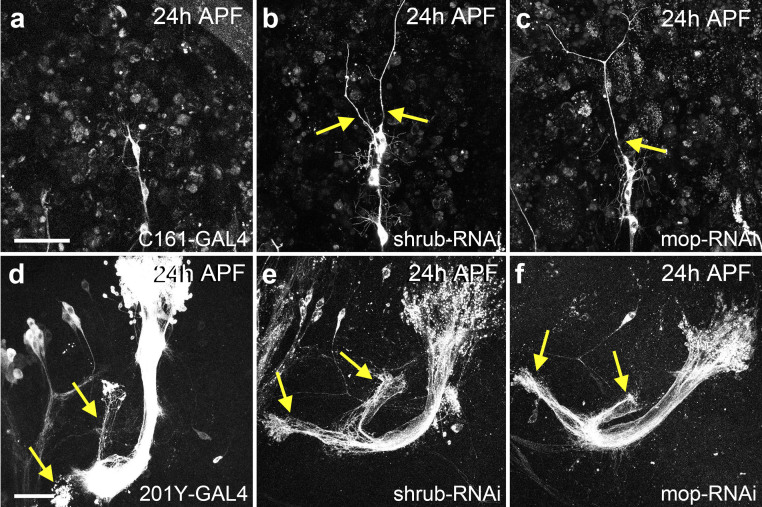
The ESCRT-III component *shrub* and accessory *mop* are required for axon and dendrite pruning. (a) C161-GAL4 labels class I da sensory neurons ddaD and ddaE that undergo remodeling; at 24 h APF the dendrites of have been pruned. (b) In class I da neurons expressing shrub-RNAi, some dendritic branches have not been severed (arrow). (c) At 24 h APF class I da neurons expressing mop-RNAi still have intact dendritic branches. (d) At 24 h APF, the axons of mushroom body γ-neurons have been pruned to the node at which they branch (arrows). (e) In γ-neurons expressing a shrub-RNAi a large number of axons fail to prune by 24 h APF (arrows). (f) γ-neurons of the mushroom body expressing mop-RNAi fail to prune their axons by 24 h APF (arrows). Scale bar = 50 μm.

To determine if ESCRT function during pruning is also conserved in other type of neurons, we focused on Shrub and Mop. We found that *shrub* is required for dendrite severing in class I da sensory neurons (Fig. 4a and b)^22^ and also for severing the axons of the mushroom body γ-neurons within the central nervous system (Fig. 4d and e)^23^. Mop-RNAi recapitulates the strong severing defect seen with *shrub* loss of function revealing a new role for *mop* in branch severing (Fig. 4c and f). We conclude that Shrub and mop are key players in pruning and that ESCRT function is required, in a cell autonomous manner, for pruning in different classes of neurons in both the peripheral and the central nervous system of *Drosophila*.

### ESCRT pruning defects are not due to early developmental disruption

**Figure 5:**
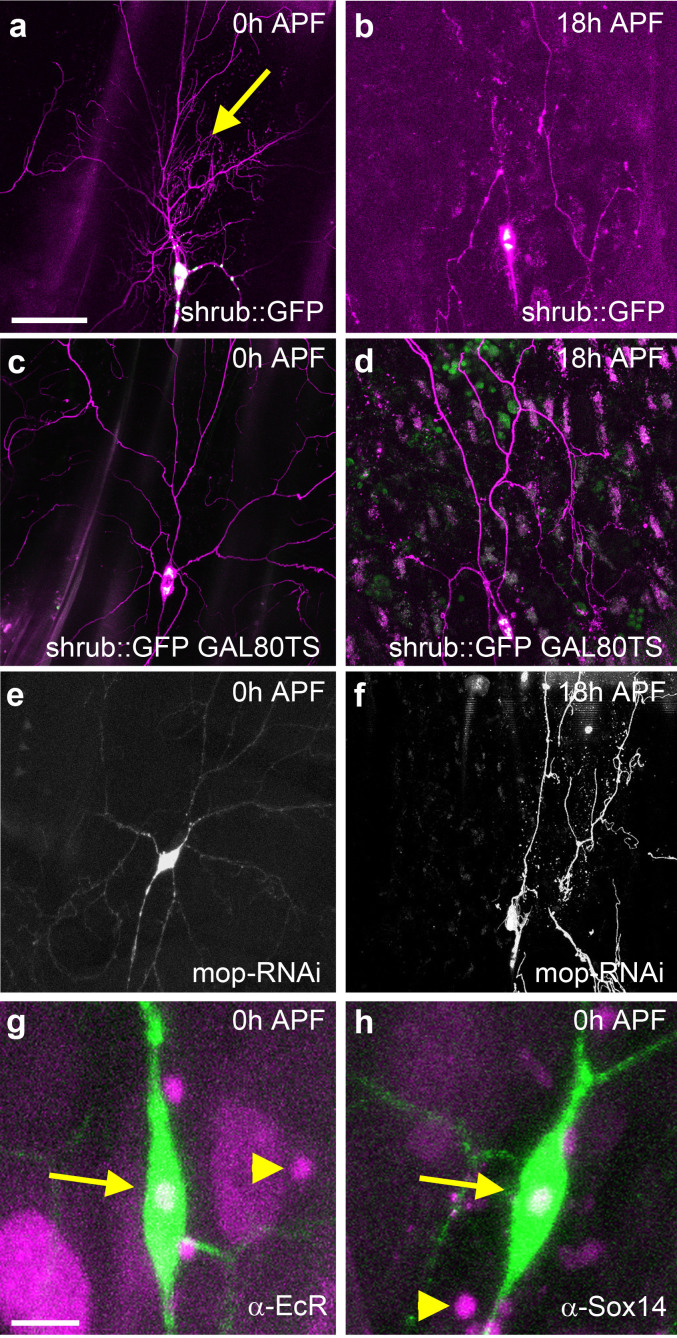
Shrub::GFP induced severing phenotypes are independent of early disruptions in class IV neuron growth. shrub::GFP acts as a dominant negative and its expression throughout larval life disrupts the morphogenesis of class IV neuron resulting in an increase of proximal branching (arrow), n = 10 (a). At 18 h APF these neurons show a robust disruption in pruning and branches fail to sever, n = 12 (b). Using a conditional GAL80^TS^ system, animals kept at the permissive temperature do not express shrub::GFP and develop wild-type arborizations, n = 16 (c). When shifted to the restrictive temperature, at 0 h APF these neurons start to express shrub::GFP and this leads to a robust severing phenotype at 18 h APF, n = 18 (d). ddaC neurons expressing mop-RNAi shows wild-type levels of branching at 0 h APF (e), whereas they exhibit a robust severing phenotype at 18 h APF (f). Animals expressing RNAi against *mop* show normal timing of expression of the ecdysone receptor gene *EcR* (g) and the HMG transcription factor *Sox14* (h) (arrow) compared to neighboring da neurons (arrow head). (a–f) Scale bar = 50 μm. (g, h) Scale bar = 10 μm.

Previous work showed that shrub loss of function disrupts the development of da neuron arborizations and leads to the eponymous bushy ‘shrub’ phenotype^15^. To determine if these early developmental defects in arbor growth could be responsible for the observed disruptions during pruning we utilized a GFP tagged Shrub (shrub::GFP) that behaves as a dominant negative^15^. This construct provided us with the opportunity to measure the level of Shrub expression through monitoring the GFP. When shrub::GFP is expressed continuously in ddaC neurons we observe both a ‘shrub’ branching phenotype at 0 h APF (Fig. 5a) as well as disruption in pruning at 18 h APF (Fig. 5b). If we delay the onset of expression shrub::GFP to late wL3 stage, the dendritic arborization is wild-type at 0 h APF (Fig. 5c) but we still find strong severing defects, n = 15 (Fig. 5d). Alongside this, we find that when mop-RNAi is expressed throughout development, the dendritic arborization of ddaC does not show a shrub-like branching phenotype at 0 h APF (Fig. 5e), but does have a robust severing defect at 18 h APF (Fig. 5f). Taken together, these data show that disruptions in pruning can be decoupled from the early developmental defects of ESCRT loss of function.

To determine whether the initiation of pruning takes place on schedule we also looked at the expression of the *Ecdysone Receptor* (*EcR*), and *Sox14*^24^,^25^, two key genes that are upstream components in the pruning cascade. We found that ddaC neurons expressing mop-RNAi had same onset and same levels of expression of both transcription factors as adjacent wild-type da neurons at 0 h APF (Fig. 5g and h).

Taken together these data show that the gating of pruning proceeds normally and that the severing defects are the result of a requirement for the ESCRTs function during pruning rather than from some earlier developmental sequela.

### How do the ESCRTs act within a pruning neuron?

Mop and Shrub, like other components of the ESCRT machinery, are required for ubiquitinated endosomal cargos to be sorted into the Multivesicular Bodies (MVB) for degradation. One possibility is that the dendrite severing phenotypes we observe are the result of a disruption in this process. To address this question we examined levels of ubiquitinated proteins in ddaC neurons under different conditions.

**Figure 6:**
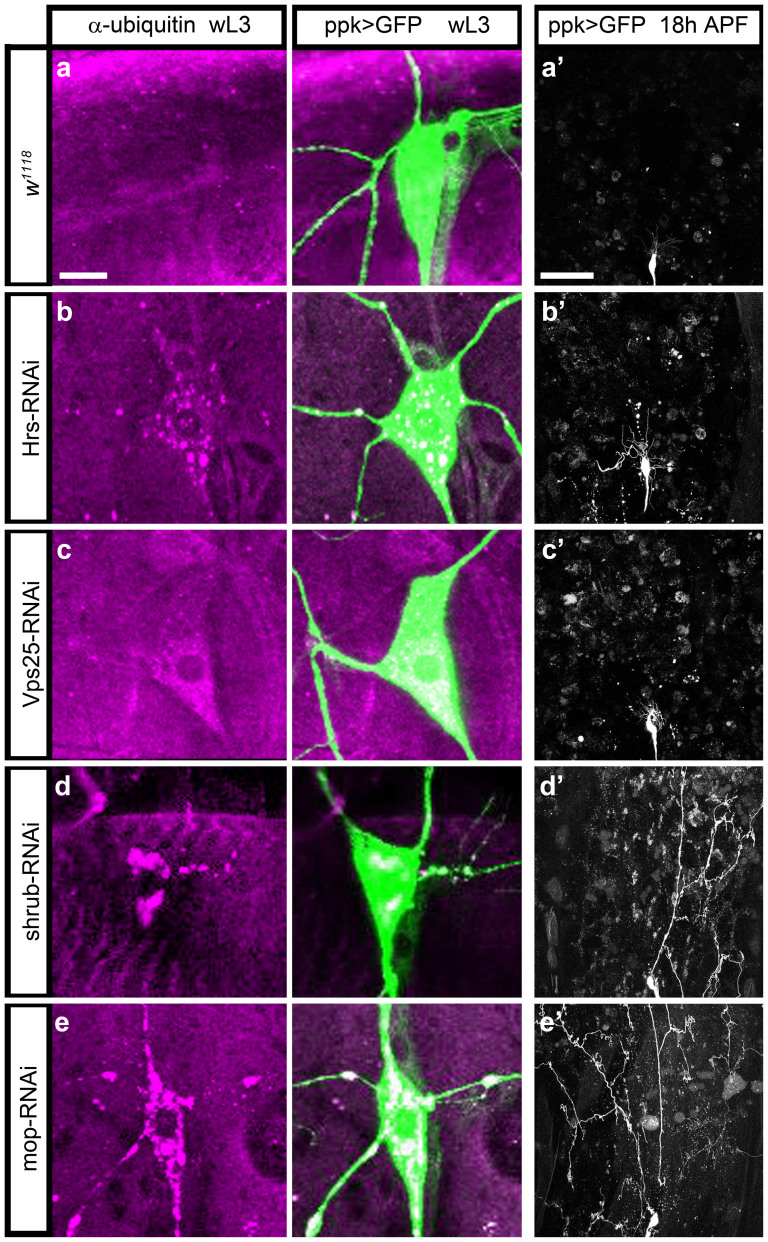
ESCRT mediated defect in MVB biogenesis is not sufficient to induce pruning dysfunction. Images of larval ddaC labeled with CD8::GFP labeled with antibody recognizing ubiquitinated proteins. *w^1118^* shows no accumulation of ubiquitinated proteins in a wandering third instar larva (wL3) (a) and no severing defect at 18 h APF (a′). An RNAi against *Hrs*, ESCRT-0, shows an accumulation of ubiquitinated proteins (b) and a mild clearance defect but no disruption of severing (b′). RNAi against *Vps25*, ESCRT-II shows an accumulation of ubiquitinated proteins (c) and no pruning defect (c′). RNAis against *shrub* and *mop* result in the accumulation of ubiquitinated proteins (d, e) and strong severing defect (d′, e′). (a–e) Scale bar = 10 μm. (a′–e′) Scale bar = 50 μm.

In wild-type neurons we found ubiquitinated proteins are rapidly degraded and are almost undetectable (Fig. 6a)^26^,^27^. We then analyzed the accumulation of ubiquitinated proteins in ddaC neurons where we had down regulated specific ESCRT subunits using RNAi and also compared their pruning. For all the subunits tested, we found a robust accumulation of ubiquitinated proteins revealing ESCRT dependent defects in MVB formation (Fig. 6b–e). With RNAi against the ESCRT-0 subunit *Hrs* we observed that ubiquitinated proteins accumulate in the cell body but show no severing defects at 18 h APF and only a very mild delay in the clearance of severed branches (Fig. 6b and b′). Similarly, when we knock down *Vps25*, we found the accumulation of ubiquitinated protein and no pruning defect (Fig. 6c and c′). In contrast, *shrub* and *mop* RNAi lead to an accumulation of ubiquitinated proteins in the ddaC neurons, as expected from their requirement in MVB biogenesis and also show robust disruptions in severing with many dendritic branches still attached at 18 h APF (Fig. 6d–e′). These data suggest that the MVB compartment may be involved in pruning but disrupting its function is not sufficient to cause the severing defects observed with *shrub* and *mop* knockdown. These data confirm the importance of the ESCRT machinery in controlling MVB biogenesis in neurons but also appear to decouple MVB cargo processing and branch severing. This raises the possibility that the ESCRTs may play an additional role in pruning that is independent of MVB biogenesis.

### Could the ESCRT machinery play a local role within dendrites during pruning?

**Figure 7:**
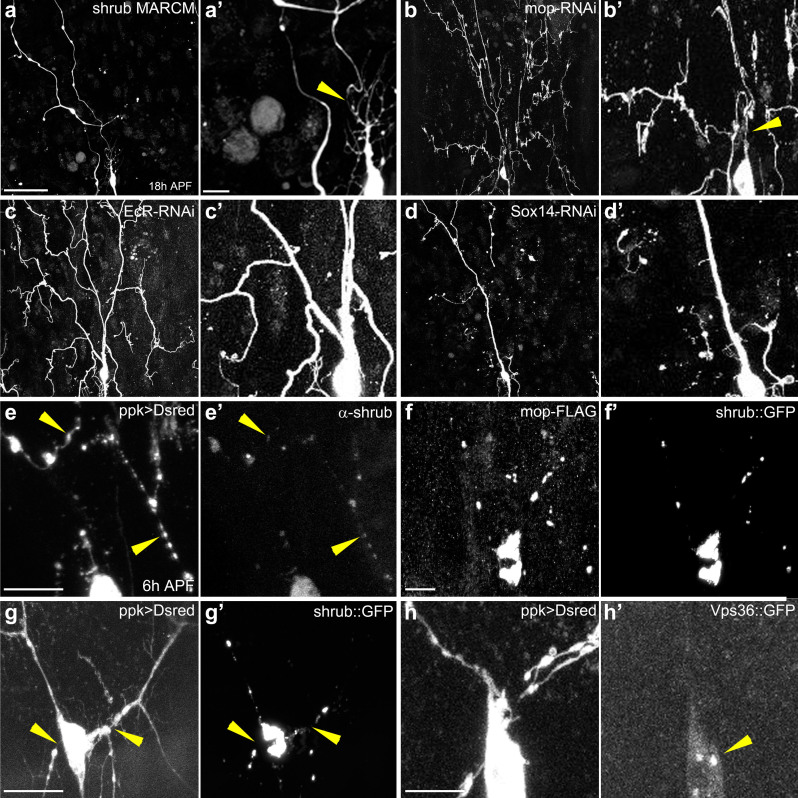
Analysis of proximal dendritic branches and localization of ESCRT components during pruning. (a and a′) ddaC homozygous MARCM clone for *shrub^4^* at 18 h APF. A very thin membrane tether attaches distal branches to the cell body (arrow head). (b and b′) ddaC expressing mop-RNAi at 18 h APF. a very thin membrane tether attaches distal branches to the cell body (arrow head). (c, c′ and d, d′) ddaC expressing Ecr-RNAi or Sox14-RNAi at 18 h APF show robust proximal dendrites with a calibre similar to that seen in prepupae. (e, e′) Antibody staining against Shrub reveals its localization within the cell body and the proximal dendrites at 6 h APF in a ddaC neuron, expressing CD8::DsRed under the control of ppk-GAL4 (arrow head). Detail shows Shrub localized into varicosities and small puncta within the proximal branches. (f, f′). Staining with an antibody against Flag reveals that mop::FLAG is strongly co-localized with shrub::GFP within the dendrites of ddaC neuron at 6 h APF. (g, g′) shrub::GFP is localized to the cell body and the proximal dendrites of ddaC neurons at 6 h APF (arrow head). (h, h′). Vps36::GFP is found in the cell body (arrow head), with no obvious localization in proximal dendrites of the ddaC at 6 h APF (a, b, c and d) Scale bar = 50 μm. (a′, a′, c′, d′ and e–h′) Scale bar = 10 μm.

Live imaging studies of pruning in ddaC neurons show that the proximal branches remodel their cytoskeleton, become thin and then sever^22^. A closer analysis of the severing defect shows that a number of clones homozygous mutant for Shrub (Fig. 7a–b) had a very thin membrane tether connecting the distal dendrites to the cell body (Fig. 7a′–b′). This change in the calibre is in stark contrast to neurons in which EcR or Sox14 function has been disrupted by RNAi, where the diameter of the proximal dendrites remain similar to those of larval neurons (Fig. 7c–d′). Following these observations, we wondered whether the ESCRT machinery could be acting locally to cut the dendritic membranes and that these very thin tethers were unresolved scission events.

To further explore the idea of a local action, we examined the localization of Shrub in ddaC neurons at 6 h APF while pruning is occurring. We found Shrub present in the cell body, as would be expected for its role in the MVB biogenesis, but also found it within the proximal dendrites (Fig. 7e–e′). The distribution of Shrub within the proximal dendrites was not homogenous, but localized to varicosities and in small puncta within the thinner parts of the dendrites, in the region where the severing events will occur (Fig. 7e–e′). We also analyzed the localization of shrub::GFP in ddaC neurons during pruning (Fig. 7g–g′) and found it mirrored the antibody staining.

From the interaction study, we hypothesized that the accessory protein Mop should be spatially and temporally localized with Shrub. Using a Flag-tagged version of Mop^21^, we observed a robust co-localization of Mop with Shrub::GFP during the early phases of ddaC pruning (Fig. 7f–f′) consistent with Mop playing a local action to recruit/stabilize Shrub and the ESCRT pruning module. In contrast to this, we found that Vps36::GFP, a member of ESCRT-II, required for MVB formation but not for pruning, was localized only within the cell body and not in the proximal dendrites (Fig. 7h–h′). Altogether, these data provide support for a local action of an ESCRT pruning module, requiring Shrub and Mop.

## Discussion

The normal functioning of the nervous system requires appropriate matching between signaling and receiving cells. This ‘matching’ of network components is achieved by progressive developmental phenomena, such as cell division and cell growth, and regressive phenomena, such as programmed cell death and pruning. Large-scale pruning, where relatively long neuron branches are removed, happens not by a distal to proximal retraction event but by a local degeneration. How such highly ordered, branch-specific auto-destruction events are orchestrated still remains unclear.

The proteins of the ESCRT family are highly conserved from yeast to human^28^ with a striking functional conservation of the cellular roles they play in multicellular organisms i.e. MVB formation and cytokinesis^9^,^28^. Here, we describe a RNAi screen to study the involvement of the ESCRT machinery during pruning. Functional knockdown within neurons of many members of this large family showed clearance or severing defects. As partial loss of function or off target effects can be an issue when using RNAi tools we used mulitiple RNAi reagents and also followed up candidates by generating MARCM clones with previously characterized null or strong alleles of the ESCRT proteins.

RNAi reagents against Hrs were not sufficient to allow us to conclude whether it has a role during pruning. The absence of any phenotype in MARCM clones with Hrs^D28^, a strong allele, alone or in combination with the null allele stam^2L-2896^ confirmed that none of the subunits of the ESCRT-0 are required for pruning^29^,^30^. Likewise, RNAi against ESCRT-II subunits Vps22, 25 were confirmed by MARCM clonal analysis, suggest that these subunits are not involved in pruning. Combined with the absence of phenotype in Vps36 RNAi, the third subunit of the ESCRT-II, these data indicate that ESCRT-II is not required for pruning. On the other hand, MARCM analysis of Vps28 and Shrub confirmed the cell autonomous requirement of ESCRT-I and -III, for pruning.

Together, we analyzed the loss of function of 17 ESCRT and ESCRT accessory proteins on neuron pruning. Our data reveal a novel combination of the ESCRT components that forms an ‘ESCRT pruning module’ necessary for branch severing. This module is composed of components of ESCRT-I, ESCRT-I together with Vps4 and the accessory proteins Mop and Ubpy. The module does not appear to require ESCRT -0 and -II function.

The loss of function of ESCRT genes led to both clearance and severing defects. The clearance defects we observed in this system could be the result of some disruption to the engulfment machinery or due to a delay in severing branches. Nevertheless, as the RNAi and MARCM clones are generated specifically in the neurons we decided to focus on the severing process. More specifically, we focused our attention on Shrub and Mop, as Shrub is a key component of the ESCRT-III scission machinery which forms a helical oligomeric filament that brings together membranes to allow scission to take place. The formation of the Shrub filament requires the assembly of different combinations of ESCRT complexes depending on the particular cellular process in question e.g. membrane repair, MVB, cytokinetic abscission and viral budding. The ESCRT-accessory proteins play a key role in recruiting and/or stabilizing these specific complexes. Our RNAi screen reveals that Mop is likely to be an important accessory protein required for pruning. We found that the *in vivo* physical interaction seen between the human ESCRT-III protein CHMP4B and the accessory protein HD-PTP^20^ is conserved between the *Drosophila* homologues, Shrub and Mop. Furthermore, our analyses of other da sensory neurons and γ-neurons of the mushroom body reveal that Shrub and Mop are required for both dendrite and axon pruning. Taken together these data suggest that the ‘ESCRT pruning module’ may be part of a universal machinery used for the structural remodeling of neurites.

A key question raised by the work is whether the disruptions to pruning we observe are due to a pleiotropic dysregulation of development or to a more specific event during pruning. The analysis of shrub::GFP and mop RNAi reveal that the pruning defect seen with ESCRT loss of function are independent from the earlier developmental disruption they can induce. Furthermore, the expression of EcR and Sox14, two markers of pruning initiation, were normal in mop RNAi neurons. These results reveal a role for the ESCRTs in ddaC neurons during metamorphosis.

How is a dysregulation of the ESCRT machinery actually disrupting pruning? Two recent papers forward the hypothesis that the ESCRT dependent disruption of pruning is through its role in MVB biogenesis and the processing of cell surface receptors^31^,^32^. Zhang *et al* showed that Vps28, Vps32 and Vps4 are required for pruning in ddaC^31^. These three ESCRT subunits belong to what we call the ESCRT pruning module, thus corroborating our findings. Our analyses show that these subunits are important in MVB formation and processing ubiquitinated proteins. Interestingly, we observed that other subunits of the ESCRT-0 and -II machinery, Hrs or Vps25, also affect MVB formation and ddaCs ability to process ubiquitinated proteins. We noticed different patterns of accumulation of the ubiquitin staining depending on the ESCRT complex analyzed. These variations could be due to the stage of MVB formation at which these subunits act i.e. Hrs at the beginning, Vps32 and Vps4 for the later steps. Importantly, we find that disruption in ESCRT-0 and -II do not lead to severing defects. This could be due to redundancy of the components, with one being able to compensate for the other, although we find severing occurs normally in the double mutant for Hrs/Stam. Another possible caveat could be that there is a perdurance of the wild-type protein within the single-cell mutant clones i.e. sufficient wild-type protein is transferred across into the homozygous mutant cell at the point of clone induction. This scenario also seems unlikely as 4 days have elapsed between the recombination event and branch severing, during which time the cell has grown in size by at least two orders of magnitude. Thus, our results point toward a decoupling between MVB formation and severing, suggesting that even though the ESCRTs may be involved in pruning through MVB biogenesis they may also be required through some other mode of action.

It has not escaped our attention that there is a striking topological similarity between neurite severing and the events taking place at cytokinetic abscission. It could be that the ESCRT pruning module is required locally to physically cut the neurites. We find that Shrub and Mop are co-localized within the proximal dendrites. This ‘local hypothesis’ could also explain the gossamer-thin tethers linking the distal dendrites to the cell body we observed in the ESCRT loss of function, a phenotype that we have never seen before in our studies of pruning. This structure is reminiscent of an unresolved intercellular bridge as seen when the ESCRTs is disrupted during cytokinetic abscission. Furthermore, there is a parallel between cytokinetic abscission and pruning in that both ESCRT-I and -III are involved but not ESCRT-II.

**Figure 8:**
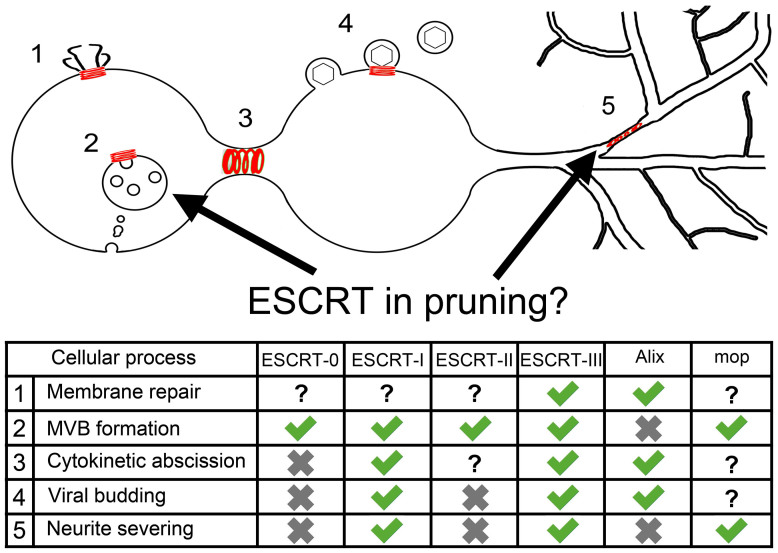
ESCRT machinery roles. Schematic summarizing the role of the ESCRT machinery in membrane scission events at different cellular locations at different times in the life of a cell. 1. Membrane repair, 2. MVB formation, 3. Cytokinetic abscission, 4. Viral budding 5. Neurite branch severing. The table summarises the deployment of ESCRT components and highlights that specific combinations of ESCRT proteins assemble depending on which membrane-cutting event is required^5^,^6^,^7^,^8^,^9^. Tick indicates that the specific ESCRT complex is involved, cross that it is not required and ? indicates that at present its role is not known.

We found it appealing that the ESCRT machinery could be deployed for a very ‘neuron-specific’ function, like pruning, as until now most studies have focused on common cellular phenomena found in all cell types (Fig. 8). Hopefully improvements in imaging techniques along with genome engineering will soon enable us to observe the dynamics and localization of shrub within the dendrites during pruning to explicitly test this ‘local hypothesis’. Future work exploring the detailed composition and assembly of this ESCRT ‘pruning module’ will provide important insights into the cellular events leading up to pruning.

## Methods

### Plasmid Construction

Shrub and the Bro domain of Mop (aa 1–389) were amplified by PCR from cDNA (pUAST-mop a gift from Jessica Treissman^21^) using primers directed to the 5′ and 3′ ends of the coding sequence. Primers contained NotI sites to allow the insertion of the PCR product into pGBKT7 (Clontech) and pVP16^33^ for the yeast two-hybrid assays. The point mutations I201D/V205D was introduced by PCR based site-directed mutagenesis. cDNA were then cloned into the phiC31 vector pUASTattB to allow directed transgenesis at two independent AttP sites, attP40 on 2L and VK00027 on 3R.

### Yeast-Two-Hybrid Assay

Yeast Y190 cells were transformed with 1 μg of each of the pGBKT7 and pVP16 constructs. Transformants were selected on media lacking tryptophan and leucine for 3 days at 30°C. Interactions were determined by β-galactosidase activity in yeast extracts as previously described^34^.

### Fly stocks

The following GAL4 driver strains were used: ppk1.9-GAL4 driver, expressed in ddaC neurons and occasionally in isolated epidermal cells; C161-GAL4, expressed in five dorsal da neurons; 201Y-GAL4, expressed in γ-MB neurons; and elav^c155^-GAL4, a general neuronal driver. For this study we used the following flies: SOP-FLP from Tadashi Uemura, *shrub^4^*, UAS-shrub::GFP and UAS-shrub-RNAi#1 from Dr Fen-Biao Gao; UAS-mop-FLAG from Dr Jessica Treismann; UAS-EcR-RNAi^CA104^; *Alix^LL05494^* (DGRC140933 Kyoto); *Hrs* ^D28^; *Hrs*^D28^,*Stam*^2L-2896^ BL41806, *Vps22^SS6^*, *Vps25^A3^* and *Vps28^B9^* from Bloomington Stock Center. The RNAi lines UAS-Hrs-RNAi-BL33900, UAS-TSG101-RNAi-BL35710, UAS-Vps25-RNAi-BL26286, UAS-Vps36-RNAi-BL38286, UAS-Vps2-RNAi-BL38995, UAS-CHMP2B-RNAi-BL38375, UAS-Vps20-RNAi-BL40894, UAS- Vps24-RNAi-BL39281, UAS-mop-RNAi-BL32916 and UAS-ubpy-RNAi-BL38982 were obtained from Bloomington Stock Center. UAS-Dicer2 on the X chromosome was used with ppk-GAL4 for our screen. The RNAi lines UAS-Vps22-RNAi-21658GD; UAS-Alix-RNAi-32047GD and UAS-Vps60-RNAi-101422KK were obtained from the Vienna Drosophila RNAi Centre. The RNAi line UAS-Vps4-RNAi-6841R1 was obtained from the National Institute of Genetics (Japan). Appropriate genotypes of both sexes were used in this study.

### Modified mosaic analysis with a repressible cell marker

For our modified mosaic analysis with a repressible cell marker (MARCM) (Lee and Luo, 1999) of da sensory neurons, clones were induced in the embryo by SOP-FLP. The following crosses were generated:

- ♀ w, elav^C155^-GAL4, UAS-RedStinger, SOP-FLP; tub-GAL80, FRT40A/CyO crossed with ♂ w; *Hrs^D28^*, FRT40A/CyO; ppk-eGFP
- ♀ w, elav^C155^-GAL4, UAS-RedStinger, SOP-FLP; tub-GAL80, FRT40A/CyO crossed with ♂ w; *Hrs^D28^*,*Stam*^2L-2896^ FRT40A/CyO; ppk-eGFP
- ♀ w, elav^C155^-GAL4, UAS-RedStinger, SOP-FLP; FRT42B, tub- GAL80/CyO crossed with ♂ w; FRT42B *shrub^4^*/CyO; ppk-eGFP
- ♀ w, elav^C155^-GAL4, UAS-RedStinger, SOP-FLP; FRT42D, tub-GAL80/CyO crossed with ♂ w; FRT42D, *Vps25^A3^*/CyO; ppk-eGFP
- ♀ w, elav^C155^-GAL4, UAS-RedStinger, SOP-FLP; FRT42D, tub-GAL80/CyO crossed with ♂ w; FRT42D, *Vps28^B9^*/CyO; ppk-eGFP
- ♀ w, elav^C155^-GAL4, UAS-RedStinger, SOP-FLP; ppk-eGFP; FRT82B, tubGAL80/TM6 b Tb Hu crossed with ♂ w; ppk-eGFP; FRT82B, *Alix^LL05494^*/Tm6 b Tb Hu
- ♀ w, elav^C155^-GAL4, UAS-RedStinger, SOP-FLP; ppk-eGFP; FRT82B, tubGAL80/TM6 b Tb Hu crossed with ♂ w; ppk-eGFP; FRT82B, *Vps22^SS6^*/Tm6b Tb Hu

All ddaC neurons were identified at 18 h after puparium formation (APF) by the expression of ppk-eGFP, MARCM clones expressed the RedStinger nuclear reporter protein. The GAL4 enhancer trap elav^C155^ expresses strongly in neurons and also at low level in other tissues (including the epidermis) allowing us to see the number and location of ‘collateral clones’ generated. The neighbouring GFP-labelled non-MARCM ddaC neurons provided very important controls for non-cell autonomous phenotypes.

### Staging of animals

Individual animals were collected at pupariation and maintained at 25°C in a Petri dish with moist filter paper. Staging was denoted as hours after puparium formation or APF.

### Immunocytochemistry

Immunocytochemistry was performed as described by Truman et al. (2004). Primary antibodies used were rabbit anti-GFP diluted 1/500 (Invitrogen), mouse anti-mop diluted 1/100 (Abcam) kindly provided by Dr Melissa Gilbert-Ross (Winship Cancer Institute of Emory University, Atlanta, USA (Gilbert et al., 2011), rabbit anti-shrub diluted 1/1000 kindly provided by Dr Fen Biao Gao and mouse anti-Ubiquitin FK2 diluted 1/1000 (Biomol) and mouse anti-Flag M2 diluted 1:500 (Sigma). Secondary antibodies used were FITC donkey anti-rabbit or anti-mouse IgG diluted 1:500 from Jackson ImmunoResearch Laboratories and Cy3-conjugated donkey anti-rat or anti-mouse IgG diluted 1:500 from Stratech Scientific.

### Imaging, image analysis and quantification

The body walls of third-instar larvae, white pre-pupae, and pupae (until 12 h APF) were imaged directly using a Zeiss LSM 510 confocal microscope. After 12 h APF, pupae were peeled out of the pupal case. Between 15–40 optical sections at 1 μm intervals were taken for each neuron, and assembled in NIH ImageJ (http://rsb.info.nih.gov/ij/). Maximum projections generated. Images were adjusted only for brightness and contrast using Adobe Photoshop (Adobe Systems).

## Author Contributions

All authors contributed to the conception and underlying ideas within the study. N.L. and M.A. performed experimental work. D.W. and N.L. prepared figures. D.W. and N.L. wrote the main manuscript text. All authors reviewed the manuscript.
